# Identifying the Biomarker Profile of Pre-Frail and Frail People: A Cross-Sectional Analysis from UK Biobank

**DOI:** 10.3390/ijerph20032421

**Published:** 2023-01-29

**Authors:** Wenying Chu, Nathan Lynskey, James Iain-Ross, Jill P. Pell, Naveed Sattar, Frederick K. Ho, Paul Welsh, Carlos Celis-Morales, Fanny Petermann-Rocha

**Affiliations:** 1 BHF Cardiovascular Research Centre, School of Cardiovascular and Medical Sciences, University of Glasgow, Glasgow G12 8TA, UK; 2 School of Health and Wellbeing, University of Glasgow, Glasgow G12 8RZ, UK; 3 Laboratorio de Rendimiento Humano, Grupo de Estudio en Educación, Actividad Física y Salud (GEEAFyS), Universidad Católica del Maule, Talca 3466706, Chile; 4 Centro de Investigación Biomédica, Facultad de Medicina, Universidad Diego Portales, Santiago 8370068, Chile

**Keywords:** frailty, aging, biomarkers, UK Biobank

## Abstract

Objective: This study aimed to compare the biomarker profile of pre-frail and frail adults in the UK Biobank cohort by sex. Methods: In total, 202,537 participants (67.8% women, aged 37 to 73 years) were included in this cross-sectional analysis. Further, 31 biomarkers were investigated in this study. Frailty was defined using a modified version of the Frailty Phenotype. Multiple linear regression analyses were performed to explore the biomarker profile of pre-frail and frail individuals categorized by sex. Results: Lower concentrations of apoA1, total, LDL, and HDL cholesterol, albumin, eGFRcys, vitamin D, total bilirubin, apoB, and testosterone (differences ranged from −0.30 to −0.02 per 1-SD change), as well as higher concentrations of triglycerides, GGT, cystatin C, CRP, ALP, and phosphate (differences ranged from 0.01 to 0.53 per 1-SD change), were identified both in pre-frail and frail men and women. However, some of the associations differed by sex. For instance, higher rheumatoid factor and urate concentrations were identified in pre-frail and frail women, while lower calcium, total protein, and IGF-1 concentrations were identified in pre-frail women and frail women and men. When the analyses were further adjusted for CRP, similar results were found. Conclusions: Several biomarkers were linked to pre-frailty and frailty. Nonetheless, some of the associations differed by sex. Our findings contribute to a broader understanding of the pathophysiology of frailty as currently defined.

## 1. Introduction

Frailty is recognized as a state of decreased reserve and diminished resilience to stressors among middle- and older-aged people, resulting from an accumulated decline in multiple physiological systems [1]. Although previous studies have recognized the relevance of identifying frailty in the population, no classification exists under the International Classification of Diseases [2]. Moreover, though the operational classifications and criteria used to define it have been widely used by the scientific community, there is not a “gold standard” definition for frailty. A systematic review published in 2020 reported that frailty is an increasingly common syndrome among adults over 60 years old [3]. In that study, the frailty phenotype prevalence ranged from 4.9% to 65.2%, while a pooled prevalence of frailty in the UK was 7.8% [3].

The World Health Organization (WHO) suggested that understanding the biological processes related to frailty and their corresponding biomarkers could be the first step in addressing this emerging geriatric syndrome [4]. The latter is directly associated with the increasing prevalence in the older population and, even if frailty is usually recognized as a geriatric syndrome, evidence has also shown a high prevalence in middle-aged people [5,6]. Awareness of the need to identify candidates’ biological markers for frailty has been increasing and some previous studies have proposed multi-marker analytical strategies to identify potential biomarkers of frailty [7]. However, a paucity of high-quality evidence still exists. 

Some potential biomarkers—including the endocrine system, metabolic process, inflammation, renal function, liver function, and cardiovascular system—have been identified [8,9,10,11,12,13,14]. In fact, a recent systematic review and meta-analyses highlighted that several metabolic (e.g., glucose), inflammatory (e.g., interleukin-6), and hematologic (hemoglobin) markers are identified in frail and also sarcopenic people [15]. These biomarkers may play essential roles in processes preceding the development of frailty owing to their relationship with aging and systematic changes [10,11,12]. Previous studies supported instructive findings regarding the underlying mechanisms of frailty; however, caution must be taken when interpreting the data given the heterogeneity resulting from small samples, inconsistent measurements, and nonstandard diagnostic criteria [4,16,17]. Considering these limitations, this study aimed to compare—by sex—the biomarker profiles of pre-frail and frail middle-aged and older-aged individuals, with non-frail individuals, using data from the UK Biobank cohort. We hypothesized that different concentrations of biomarkers will be observed between frail, pre-frail, and non-frail groups.

## 2. Materials and Methods

This cross-sectional study used baseline data from the large UK Biobank prospective cohort study (www.ukbiobank.co.uk accessed on 2 December 2022). UK Biobank is an open-access and largescale, general population cohort study containing in-depth health information. From 2006 to 2010, more than half a million men and women aged 37 to 73 years were recruited from 22 assessment centers across England, Wales, and Scotland (5.5% response rate) [18]. All participants completed a touch-screen questionnaire, had physical measurements taken, and provided blood, urine, and saliva samples at baseline [18]. At baseline, the average age was 56.5 years (8.1 years) and 54.4% of the sample were women. Most participants had a white background (94.6%), and around 33% of the sample had a college or University degree.

### 2.1. Frailty Definition

Weight loss, exhaustion, physical activity, walking speed, and grip strength are the five criteria used to define the frailty phenotype. A modified version of the original frailty phenotype [19], however, was used in this study to fit the available data in UK Biobank [5,20]. Weight loss, tiredness/exhaustion, gait speed, and grip strength were derived following the same methodology previously described by Hanlon et al. [5]. Physical activity, in turn, was collected using the International Physical Activity Questionnaire (IPAQ) short form. This adapted form has been previously used and published as described elsewhere [6,20]. More information is also available in Supplementary Tables S1 and S2. Using the five criteria, participants were classified as frail if they met three or more criteria, pre-frail if they met one or two criteria and non-frail if they met none of the criteria. 

### 2.2. Biomarkers

In total, 30 biomarkers were available in UK Biobank initial assessment and were included in this study. These biomarkers were C-reactive protein (CRP), alkaline phosphate (ALP), phosphate, gamma-glutamyltransferase (GGT), aspartate aminotransferase (AST), rheumatoid factor, alanine aminotransferase (ALT), lipoprotein A, triglycerides, urate, urea, hemoglobin A1c (HbA1c), testosterone, oestradiol, glucose, apolipoprotein A1 (apoA1), apolipoprotein B (apoB), sex hormone-binding globulin (SHBG), insulin-like growth factor 1 (IGF-1), direct bilirubin, total bilirubin, low-density lipoprotein (LDL) cholesterol, high-density lipoprotein (HDL) cholesterol, total cholesterol, total protein, calcium, albumin, vitamin D, creatinine, and cystatin C (Table 1). These 30 biomarkers were analyzed from blood (40–50 mL) samples at baseline [21]. The square root of the lowest and the highest detectable limits was used to impute samples outside the detectable ranges [22]. In addition, to evaluate the kidney function among pre-frail and frail individuals, we also calculated an estimated glomerular filtration rate using cystatin C-based equations (eGFRcys). This approach was used since a previous paper showed eGFRcys to be more strongly associated with adverse outcomes than traditional eGFRcr or eGFRcr-cys [23]. Therefore, the final number of biomarkers included was 31. 

### 2.3. Covariates 

Age, deprivation, ethnicity, body mass index (BMI), total sedentary time, sleeping time, processed and red meat consumption, fruit and vegetable intake, smoking, alcohol consumption, and morbidity count, as well as medication, were included as covariates in the model. Age was calculated from the date of birth at baseline assessment. Sex was self-reported. Area-based social deprivation was derived from the postcode of residence using the Townsend index [38]. BMI was calculated from measured height and weight using the standard formula [39]. 

Time spent on sedentary activities (such as driving, watching television, and using a computer) and sleeping time were self-reported [40]. The frequency of processed meat, red meat, and fruit and vegetable consumption was self-reported at baseline [41]. Smoking status was self-classified as never, previous, or current. Frequency of alcohol intake was classified as almost daily, 3–4 times a week, 1–2 times a week, 1–2 times a month, special occasions, or never [20]. Prevalent morbidity was ascertained during a nurse-led interview at baseline. Morbidity count was derived from 43 long-term conditions (LTCs) as described elsewhere [42] and classified as 0 or ≥1. Medication for insulin and cholesterol was self-reported using the following question “do you regularly take any of the following medications?” More detailed information on the UK Biobank protocol can be found online (http://www.ukbiobank.ac.uk/wp-content/uploads/2011/11/UK-Biobank-Protocol.pdf accessed on 2 December 2022).

### 2.4. Statistical Analyses

A summary of descriptive characteristics was first conducted by sex and frailty status. The numerical variables were presented as means with standard deviation, and the categorical variables were presented as frequencies and percentages. 

In this study, biomarkers were expressed in two formats: as raw measurement units to identify clinically relevant differences across frailty categories and as sex-specific z-scores (per 1-SD increase) to allow comparisons between biomarkers. Using these two formats, the biomarker profile of pre-frail and frail individuals by sex was independently investigated using multiple linear regression. Results are presented as regression coefficients (β-coefficient) with their respective 95% confidence intervals (CIs). Non-frail individuals were used as the reference group. 

Our multivariates with adjusting covariates were run by sex, adjusting for the following: age; deprivation index; ethnicity; smoking status; dietary intake of red meat, processed meat, and fruit and vegetable; alcohol status; sedentary time; sleeping time; BMI; medication; and morbidity count. These covariates were taken into account for their potential effects on both the biomarkers and frailty status. In addition, all analyses were performed excluding people who self-reported drinking more than 14 units of alcohol/week using the methodology reported by Jani et al. [43]. In addition, a sensitivity analysis was performed where the model was additionally adjusted for CRP when this was not the biomarker of interest. 

The statistical analyses were performed using Stata 17 (StataCorp, College Station, TX, USA). Statistical significance was defined as a *p*-value < 0.05. Only participants with full data available to derive the exposure variable (frailty status) and covariates were included in the analyses.

### 2.5. Ethics Approval

The UK Biobank cohort analysis was approved by the Northwest Multi-Centre Research Ethics Committee (approval number: 11/NW/0382). All participants gave written informed consent to participate in the UK Biobank cohort. The study protocol is available online (http://www.ukbiobank.ac.uk accessed on 2 December 2022 ).

## 3. Results

The main characteristics of the study population are presented in Table 2, organized by sex and frailty status. Of nearly half a million participants in UK Biobank, 202,537 participants had data available on the exposure and covariates and were, therefore, included in this cross-sectional analysis. In summary, the prevalence of pre-frailty and frailty was higher in women than men (50.9% and 5.1% vs. 48.1% and 4.2%, respectively). Independent of sex, and compared to non-frail participants, both pre-frail and frail participants were older, more likely to be deprived and current smokers, and tended to have a higher BMI. However, they were less likely to drink alcohol more than three times a week (Table 2). 

**Figure 1:**
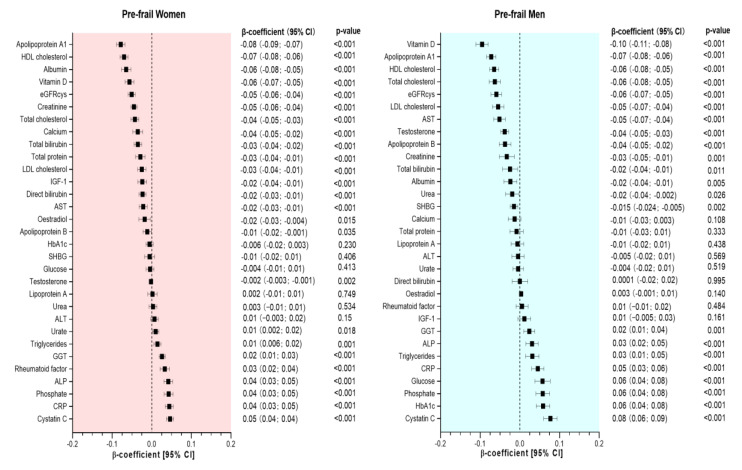
Association between biomarkers and pre-frailty by sex. Data presented as β-coefficient and its 95% CI. Non-frail individuals were considered as the reference group in each case. All analyses were adjusted for age, deprivation, BMI, smoking status, sleeping time, total sedentary time, morbidity count, medication, and dietary intake (alcohol, red meat, processed meat, fruit and vegetable intake).

**Figure 2:**
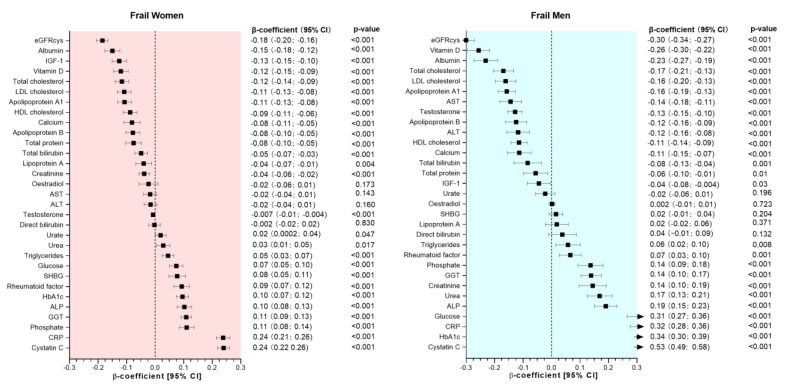
Association between biomarkers and frailty by sex. Data presented as β-coefficient and its 95% CI. Non-frail individuals were considered as the reference group in each case. All analyses were adjusted for age, deprivation, BMI, smoking status, sleeping time, total sedentary time, morbidity count, medication, and dietary intake (alcohol, red meat, processed meat, fruit and vegetable intake).

Associations between frailty status (both pre-frail and frail) and sex-standardized biomarkers are presented in Figure 1 and Figure 2. After adjusting for covariates, 25 and 22 of the 31 biomarkers (including eGFRcys) were associated with pre-frailty in women and men, respectively (Figure 1). In comparison, 27 and 26 biomarkers were associated with frailty in women and men, respectively (Figure 2).

Compared to non-frail women, those classified as pre-frail had lower concentrations of 17 biomarkers. The largest differences were observed for apoA1, HDL cholesterol, albumin, vitamin D, eGRFcys, creatinine, total cholesterol, calcium, total bilirubin, total protein, LDL cholesterol, IGF-1, direct bilirubin, AST, oestradiol, apoB, and testosterone, with β-coefficients ranging from −0.08 to −0.002 units of SD. Conversely, pre-frail women had higher concentrations on 8 biomarkers, including urate, triglycerides, GGT, rheumatoid factor, ALP, phosphate, CRP, and cystatin C (differences ranging from 0.01 to 0.05 per 1-SD change) (Figure 1). Compared to men without frailty, pre-frail men had higher concentrations on 8 biomarkers, including GGT, ALP, triglycerides, CRP, glucose, phosphate, HbA1c and cystatin C (differences ranged from 0.02 to 0.08 per 1-SD change), and lower concentrations (in descending order) of vitamin D, apoA1, HDL cholesterol, total cholesterol, eGFRcys, LDL cholesterol, AST, testosterone, apoB, creatinine, total bilirubin, albumin, urea, and SHBG (Figure 1).

Frail women had lower concentrations of 15 biomarkers, including eGFRcys, albumin, IGF-1, vitamin D, total cholesterol, LDL cholesterol, apoA1, HDL cholesterol, calcium, apoB, total protein, total bilirubin, lipoprotein A, creatinine, and testosterone; differences in β-coefficients ranged from −0.18 to −0.007 per 1-SD change. They also had higher concentrations of urate, urea, triglycerides, glucose, SHBG, rheumatoid factor, HbA1c, ALP, GGT, phosphate, CRP, and cystatin C with β-coefficients ranging from 0.02 to 0.24 per 1-SD change (Figure 2). In contrast, frail men had significantly lower concentrations of eGFRcys, vitamin D, albumin, total cholesterol, LDL cholesterol, apoA1, AST, testosterone, apoB, ALT, HDL cholesterol, calcium, total bilirubin, total protein and IGF-1. Higher concentrations of triglycerides, rheumatoid factor, phosphate, GGT, creatinine, urea, ALP, glucose, CRP, HbA1c, and cystatin C were observed in frail men compared to those who were non-frail (Figure 2). 

Biomarkers expressed in their raw measurement units are presented in Supplementary Tables S3 and S4. Finally, when analyses were further adjusted for CRP, similar patterns were observed in pre-frail and frail women and men (Supplementary Tables S5 and S6). 

## 4. Discussion

The main findings of this study highlighted that frailty and pre-frailty were associated with higher concentrations of triglycerides, GGT, cystatin C, CRP, ALP, and phosphate both in men and women. Higher rheumatoid factor and urate concentrations were also identified in pre-frail and frail women; higher glucose and HbA1c concentrations in frail women and pre-frail and frail men, while higher urea levels in frail men and women. In contrast, our findings identified that both pre-frailty and frailty were also associated with lower levels of apoA1, total, LDL, and HDL cholesterol, albumin, eGFRcys, vitamin D, total bilirubin, apoB, and testosterone in women and men. Lower calcium, total protein, and IGF-1 concentrations were observed in pre-frail women and frail women and men; low creatinine levels in both pre-frail men and women and frail women, while lower AST levels were found in pre-frail and frail men.

Since several pathophysiological processes across multiple organ systems might be related to the risk of frailty, the corresponding biomarkers were proposed to influence frailty phenotypes [4]. Our findings are discussed in several sections regarding the related biological processes, including the endocrine system, metabolic process, inflammation, renal function, liver function, and cardiovascular system. 

### 4.1. Liver Function

Frailty—particularly its physical aspect—has recently been investigated in chronic liver disease [30,44]. A longitudinal study of men aged 70 years or older found that participants with lower ALT concentrations were more likely to be frail, with GGT and AST determined as factors that might be influencing ALT activity [30]. The investigators suggested that changes in the activity of these circulating enzymes—such as AST, ALT, and GGT—have potential value as biomarkers of frailty [30]. Another study identified that abnormal serum albumin and total bilirubin concentrations were associated with an increased risk of liver disease [31]. During chronic inflammation, such as frailty, the liver produces several acute-phase reactants. Albumin is a negative acute-phase protein that decreases its synthesis to save amino acids for producing positive acute-phase proteins more effectively [15]. Consistent with these studies, our study reported that, in both sexes, pre-frail and frail adults had higher levels of ALP, GGT, and significantly lower serum albumin and total bilirubin concentrations. Low AST and ALT concentrations have been recognized as independent risk factors for frailty [30,32]. Therefore, it is not surprising that lower AST concentrations were observed in pre-frail individuals and frail men while lower ALT only observed in frail men. In frail women, lower AST and ALT levels were also identified, but values were non-significant. 

### 4.2. Renal Function 

Creatinine is an indicator of both renal function and muscle mass changes (as a product of degraded creatine phosphate in muscles) [24]. Kidney disease has been independently linked to physiological changes that may predispose to frailty [26]. Due to the relationship between low muscle mass and creatinine, this biomarker could be associated with weight loss and physical inactivity, which are part of the frailty criteria [36]. Cystatin C could be another factor related to renal function, owing to its function of removing metabolic waste products and its association with kidney disease [36]. Our study contrasted with previous findings in observing higher creatinine concentrations among frail men [36]. However, in pre-frail and frail women and men, lower eGFRcys concentrations were identified, which agrees with a previous study that identified frail individuals had worse kidney function [36]. In that study, frailty and eGFRcys were strongly associated [36]. 

On the other hand, our results indicated that lower and higher urea concentrations were observed in pre-frail men and both frail men and women, respectively. Lower urate concentrations were found in both pre-frail and frail women. These findings were unexpected because a previous study reported significantly lower urate concentrations among both men and women with low skeletal muscle mass [45]. Even if a correlation between muscle mass and frailty has been previously confirmed, the different correlations by gender may be associated with hormonal differences. A cross-sectional study highlighted that estrogen promotes uric acid (UA) secretion, resulting in elevated UA levels in postmenopausal women, potentially contributing to the significant correlation between urate and muscle mass among the female population in general [46].

### 4.3. Endocrine System

Hormones that modulate the musculoskeletal system are of particular interest due to the phenotypic changes in frailty linked to muscle mass and strength losses [4]. In our study, a lower IGF-1 concentration was associated with frailty in men and women and pre-frail women. This finding is in line with a previous study which suggested that a lower IGF-1 concentration had a strong correlation with frailty [24]. Our study also identified lower vitamin D concentrations in frail and pre-frail individuals. The latter is consistent with the previous study [47]. However, vitamin D cannot be assumed to be causal of frailty because the temporality of relationships cannot be investigated in cross-sectional studies. Lower vitamin D concentrations may be an indicator of frailty. Conversely, frailty could reduce outdoor physical activity and, therefore, exposure to sunlight, resulting in reduced production of vitamin D (reverse causation [48]).

### 4.4. Chronic Inflammation 

A previous study suggested an important role of inflammation in the development of frailty, based on the catabolic effects of pro-inflammatory cytokines on muscles [11]. A negative correlation between CRP and the rate of skeletal muscle protein synthesis was reported by Toth et al. [27]. Therefore, it is unsurprising that elevated CRP concentrations were associated with frail and pre-frail status in both sexes. Considering the role of inflammation in pre-frail and frail individuals, we performed a sensitivity analysis where the analyses were further adjusted for CRP. Yet, after the adjustment, similar results were observed (Tables S3 and S4). Independently of senescence and disease severity, frailty is more prevalent in patients with rheumatoid disease, owing to chronic inflammation [28]. Likewise, our findings suggested that the rheumatoid factor was one of the significant biomarkers identified in pre-frail women as well as men and women with frailty.

### 4.5. Metabolic Process

Abnormal glucose responses, such as higher HbA1c and glucose concentrations, might be associated with a higher risk of frailty by affecting phenotypes, including weight loss, handgrip weakness, and slow gait speed [4]. Except for pre-frail women, higher glucose and HbA1c concentrations were observed in our study. In contrast, another study reported a U-shape association between glucose concentrations and the risk of frailty among older adults with diabetes [17]. However, that study could have been influenced by reverse causality as glucose levels often decline when people are sicker or especially have worse kidney function [17].

### 4.6. Cardiovascular System

A strong relationship between frailty and cardiovascular diseases, such as heart failure and myocardial infarction, has been reported in the literature [33]. One of the explanations is that cardiovascular diseases limit physical activity and decrease functional capability [33]. Lower concentrations of total, LDL, and HDL cholesterol, as well as higher triglyceride levels, were associated with frailty in previous studies [16,35,49]. These findings were also reported in our study among pre-frail and frail people. Even if these results may be surprising, changes in plasma lipid levels are well-known in the acute-phase response or are associated with malnutrition [50]. Both conditions could be presented in pre-frail and frailty people. Moreover, lower concentrations of less used lipid variables (including lower apoA1 and apoB concentrations) were associated with frailty in previous studies [51,52], which agrees with our findings. By contrast, our study reported that lipoprotein A was only significantly related to frail women. Consistent with our findings, another study confirmed that elevated lipoprotein A concentration was not associated with an increased risk of coronary artery disease in a population over 65 years [53].

### 4.7. Nutritional Markers

A systematic review of clinical intervention studies concluded that many frailty phenotypes, such as cognitive and physical function impairments, have been linked to malnutrition [54]. As a nutritional marker, circulating calcium has a key role in various physiological processes, including neuronal transmission, immune cell activation, bone health maintenance, and muscle contraction, which are related to underlying mechanisms for frailty [54]. It was also pointed out that decreased protein intake was associated with weight loss, which may further lead to a higher prevalence of frailty [37]. Therefore, it is not surprising that reduced calcium and protein concentrations were observed in both pre-frail and frail men and women in our study

### 4.8. Strength and Limitations

This is the largest cross-sectional study to investigate the association between frailty and a wide range of biomarkers. Additionally, using data from UK Biobank allowed us to adjust our analyses for a considerable number of covariates. Nonetheless, our results should be interpreted with caution since they are not exempt from limitations. Firstly, our sample was a relatively young population compared to previous studies recruiting participants aged over 65 years [1,16]. Secondly, due to the non-probability sample from the UK Biobank study, our study reported a lower prevalence of frailty (4.8%) than the UK average (7.8%) [3]. Therefore, summary statistics should not be generalized. Thirdly, our study used an adapted frailty version with a combination of self-reported questionnaires and objective measures [5,20]. Therefore, reporting bias may lead to an under-or over-estimation of specific criteria, such as gait speed and weight loss, owing to the participants’ unclear understanding of the questions and unclear recall. However, there is no reason to believe this would introduce systematic errors concerning the biomarkers measured. Several components of the frailty criteria also varied from the original frailty phenotype [19]. For instance, UK Biobank had available data on weight loss only, not the reason for it, which may result in an underestimate of the real association with this indicator because it may include participants who intentionally lost weight. Furthermore, from the data obtained, it is impossible to determine the total amount of weight lost by individuals, which is likely to contribute significantly to the risk of frailty. Fourthly, biomarkers were collected through a random blood sample and not after fasting. Therefore, nondifferential misclassification might be an issue for some biomarkers, such as glucose. Finally, our study cannot show temporal relationships between frailty and these biomarkers due to its cross-sectional design. However, the aim of this study was to characterize and compare the biomarker profile rather than demonstrate causality.

## 5. Conclusions

Using baseline data from the UK Biobank study, we highlighted that higher concentrations of triglycerides, GGT, cystatin C, CRP, ALP, and phosphate, as well as lower concentrations of apoA1, total, LDL and HDL cholesterol, albumin, eGFRcys, vitamin D, total bilirubin, apoB, and testosterone were identified both in pre-frail and frail men and women. Despite that, some associations differed by sex, as shown in the results section. Our findings might contribute to a better understanding of the possible biological processes occurring among people with frailty by analyzing a considerable number of biomarkers linked to the development of frailty. This study also provides insights into a novel method for monitoring the development of frailty using these biological profiles. Future longitudinal studies should be conducted to investigate the correlation between frailty and changes in biomarkers over an extended period. Based on the biomarkers identified in this study, future research should explore whether such biomarkers could be used to identify those at high risk of frailty early.

## Figures and Tables

**Table 1:** Biomarkers and their roles in frailty/aging.

Biomarkers (Unit)	Classification	Role in Frailty/Aging
Phosphate (mmol/L)	Endocrine system	Endocrine disturbances, such as abnormal levels of phosphate, could be linked to frailty by muscle mass, bone growth, and strength losses [4,24].
Testosterone (nmol/L)	Endocrine system	Muscle strength. Bone mineral density. Impaired mobility [25].
SHBG (nmol/L)	Endocrine system	Type 2 diabetes. Weight loss, exhaustion, and physical activity [25].
Oestradiol (pmol/L)	Endocrine system	Declined oestradiol is associated with grip strength which is one of the indicators of frailty [1].
IGF-1 (mmol/L)	Endocrine system	IGF-1 is associated with a higher risk of fracture, heart failure, and mortality which may predispose to frailty [26].
Vitamin D (nmol/L)	Endocrine system	Muscle mass and strength loss [4]. High level of Vitamin D was related to the risk of frailty progression [24].
CRP (mg/L)	Inflammation	Influencing the skeletal muscle protein synthesis rate, CRP is linked to low muscle mass and strength [27].
Rheumatoid factor (IU/mL)	Inflammation	Rheumatic disease. Chronic inflammation contributes to the development of frailty [28].
ALP (U/L)	Liver function	ALP could influence bone disorder, muscle mass, strength, and physical performance [29].
GGT (U/L)	Liver function	GGT correlated with ALT activity, which can reflect hepatic origins and is related to frailty [30].
ALT (U/L)	Liver function	Age-related biomarker. Nonalcoholic fatty liver disease [30].
AST (U/L)	Liver function	AST also correlated with ALT activity, which can reflect hepatic origins and related to frailty [26,30].
Direct bilirubin (μmol/L)	Liver function	Bilirubin is linked to a higher risk of liver disease, which is associated with energy metabolic disorders [31].
Total bilirubin (μmol/L)	Liver function	Bilirubin is linked to a higher risk of liver disease, which is associated with energy metabolic disorders [31].
Albumin (g/L)	Liver function	Hypoalbuminemia is the result of malnutrition which is associated with frailty [32].
ApoA1 (g/L)	Cardiovascular system	ApoA is a biomarker of cardiovascular function. Frailty can be accelerated by cardiovascular disease (CVD), with the cumulative sharing burden of risk factors [33].
ApoB (g/L)	Cardiovascular system	ApoB is a biomarker of cardiovascular function. Frailty can be accelerated by cardiovascular disease (CVD), with the cumulative sharing burden of risk factors [33].
Total cholesterol (mmol/L)	Cardiovascular system	Cholesterol can reflect cardiovascular function. Patients with CVD were limited to engage physical activity; thus, their functional capability declined [33].
LDL cholesterol (nmol/L)	Cardiovascular system	Vascular and all-cause mortality. Coronary heart disease and CVD [34].
HDL cholesterol (mmol/L)	Cardiovascular system	HDL, as a biomarker of cardiovascular disease, is associated with aging and all-cause mortality [34].
Triglycerides (mmol/L)	Cardiovascular system	Cardio-metabolic disease. Activities of daily living decline [35].
Lipoprotein A (nmol/L)	Cardiovascular system	Cholesterol-rich particles and CVD. Lipoprotein was defined as the indicator of cardiovascular disease, which shared pathophysiological pathways with frailty [33].
Cystatin C (mg/L)	Renal function	Cystatin C is a biomarker of kidney disease which has been independently linked to physiological changes that may predispose to a higher risk of frailty [26,36].
Urate (μmol/L)	Renal function	Biomarker of renal function. Decreased urate was significantly associated with low skeleton muscle [36].
Urea (mmol/L)	Renal function	Biomarker of kidney disease. It has been independently linked to physiological changes that may predispose to a higher risk of frailty [26,36].
Creatinine (μmol/L)	Renal function	Owing to the association between creatinine and muscle mass, it could be linked to weight loss and physical inactivity, which are part of the frailty phenotype [26].
HbA1c (mmol/mol)	Metabolic biomarker	Increased levels of HbA1c might negatively influence lean body mass [4].
Glucose (mmol/L)	Metabolic biomarker	Type 2 diabetes. Affecting weight loss, handgrip, and slow gait speed [9].
Calcium (mmol/L)	Nutritional biomarker	Lower extremity lean mass and muscle strength [24].
Total protein (g/L)	Nutritional biomarker	A parameter of nutritional status. Decreased protein is associated with weight loss and may further lead to a higher risk of frailty [37].

g/L = gram per liter; mg/L = milligrams per liter; mmol/L = millimoles per liter; nmol/L = nanomoles per liter; U/L = units per liter; μmol/L = micromoles per liter; IU/L = international units per milliliter.

**Table 2:** Baseline characteristics by sex and frailty category.

	Women (137,376)			Men (65,161)		
	No Frail	Pre-Frail	Frail	No Frail	Pre-Frail	Frail
Sociodemographic						
Total, n (%)	60,389 (44.0)	69,989 (50.9)	6998 (5.1)	31,078 (47.7)	31,332 (48.1)	2751 (4.2)
Age (years), mean (SD)	56.1 (8.09)	57.0 (8.02)	57.8 (7.74)	56.7 (8.4)	57.2 (8.39)	58.5 (7.83)
Deprivation, mean (SD)	−1.7 (2.84)	−1.2 (3.06)	−0.1 (3.47)	−1.6 (2.94)	−1.1 (3.24)	0.5 (3.64)
Ethnicity						
White, n (%)	57,716 (95.6)	65,172 (93.1)	6002 (85.8)	29,270 (94.2)	28,166 (89.9)	2263 (82.3)
South Asian, n (%)						
Black, n (%)						
Chinese, n (%)						
Others, n (%)	2673 (4.4)	4817 (6.9)	996 (14.2)	1808 (5.8)	3166 (10.1)	488 (17.7)
Anthropometric						
BMI (kg/m^2^), mean (SD)	25.6 (4.22)	27.5 (5.20)	30.9 (6.7)	26.7 (3.8)	27.9 (4.42)	29.8 (5.67)
Fitness and lifestyle						
Total sedentary behavior (h/day), mean (SD)	4.4 (1.84)	4.7 (2.01)	5.1 (2.46)	5.1 (2.29)	5.4 (2.49)	6.0 (2.96)
Sleeping time (h/day), mean (SD)	7.2 (1.05)	7.1 (1.25)	7.0 (1.74)	7.1 (1.0)	7.1 (1.21)	7.1 (1.78)
Processed meat (times/week), mean (SD)	1.5 (0.99)	1.6 (1.02)	1.7 (1.11)	2.0 (1.08)	2.1 (1.11)	2.1 (1.22)
Red meat (times/week), mean (SD)	1.9 (.99)	1.9 (1.35)	2.0 (1.51)	2.1 (1.39)	2.1 (1.49)	2.1 (1.65)
Fruit & Vegetables (grams/day), mean (SD)	376.0 (189.34)	364.1 (196.78)	356.6 (215.83)	333.4 (206.19)	327.5 (223.03)	322.7 (246.94)
Smoking status, frequency (%)						
Never	40,008 (66.3)	44,703 (63.9)	4181 (59.8)	19,080 (61.4)	18,002 (57.5)	1195 (43.4)
Previous	16,770 (27.8)	20,187 (28.9)	2002 (28.6)	9659 (31.1)	10,365 (33.1)	1097 (39.9)
Current	3611 (6.0)	5009 (7.3)	815 (11.7)	2339 (7.5)	2965 (9.5)	459 (16.7)
Alcohol intake, frequency (%)						
Almost daily	4312 (7.1)	3776 (5.4)	184 (2.6)	1610 (5.2)	1412 (4.5)	76 (2.8)
3–4 times a week	13,097 (21.7)	11,563 (16.5)	564 (8.1)	5100 (16.4)	4098 (13.1)	178 (6.5)
1–2 times a week	22,333 (37.0)	23,859 (34.1)	1672 (23.9)	13,233 (42.6)	11,995 (38.3)	726 (26.4)
1–3 times a month	3829 (6.3)	4996 (7.1)	457 (6.5)	2.362 (7.6)	2645 (8.4)	215 (7.8)
Special occasions only	10,720 (17.8)	16,054 (22.9)	2306 (33.0)	5032 (16.2)	5925 (18.9)	745 (27.1)
Never	6098 (10.1)	9741 (13.9)	1815 (25.9)	3741 (12.0)	5257 (16.8)	811 (29.5)
Morbidity count, frequency (%)						
0	25,075 (41.5)	21,057 (30.1)	883 (12.6)	13,017 (41.9)	9579 (30.6)	283 (10.3)
≥1	35,314 (58.5)	48,932 (69.9)	6115 (87.4)	18,061 (58.1)	21,753 (69.4)	2468 (89.7)
Medication, n (%)						
No	54,902 (90.9)	59,664 (85.3)	5039 (72.0)	25,451 (81.9)	23,033 (73.5)	1482 (53.9)
Yes	5487 (9.1)	10,325 (14.8)	1959 (28.0)	5627 (18.1)	8299 (26.5)	1269 (46.1)

## Data Availability

All UK Biobank information is available online on the webpage www.ukbiobank (accessed on 2 December 2022). Data access are available through applications. This research was conducted using the application number 7155.

## Supplementary Materials

The following supporting information can be downloaded at: https://www.mdpi.com/article/10.3390/ijerph20032421/s1, Table S1. Modified frailty criteria; Table S2. Cut-off points for grip strength criterion for frailty; Table S3. Biomarkers by frailty status (Women); Table S4. Biomarkers by frailty status (Men); Table S5. Associations between biomarkers and frailty categories in women; Table S6. Associations between biomarkers and frailty categories in men.

## References

[1] Schaap L.A., Pluijm S.M.F., Smit J.H., Van Schoor N.M., Visser M., Gooren L.J.G., Lips P. (2005). The association of sex hormone levels with poor mobility, low muscle strength and incidence of falls among older men and women. Clin. Endocrinol..

[2] Rockwood K., Hogan D.B., MacKnight C. (2000). Conceptualisation and Measurement of Frailty in Elderly People. Drugs Aging.

[3] Majid Z., Welch C., Davies J., Jackson T. (2020). Global frailty: The role of ethnicity, migration and socioeconomic factors. Maturitas.

[4] Wang J., Maxwell C.A., Yu F. (2019). Biological Processes and Biomarkers Related to Frailty in Older Adults: A State-of-the-Science Literature Review. Biol. Res. Nurs..

[5] Hanlon P., Nicholl B.I., Jani B.D., Lee D., McQueenie R., Mair F.S. (2018). Frailty and pre-frailty in middle-aged and older adults and its association with multimorbidity and mortality: A prospective analysis of 493 737 UK Biobank participants. Lancet Public Health.

[6] Petermann-Rocha F., Lyall D.M., Gray S.R., Esteban-Cornejo I., Quinn T.J., Ho F.K., Pell J.P., Celis-Morales C. (2020). Associations between physical frailty and dementia incidence: A prospective study from UK Biobank. Lancet Healthy Longev..

[7] Calvani R., Picca A., Marini F., Biancolillo A., Gervasoni J., Persichilli S., Primiano A., Coelho-Junior H.J., Cesari M., Bossola M. (2021). Identification of biomarkers for physical frailty and sarcopenia through a new multi-marker approach: Results from the BIOSPHERE study. GeroScience.

[8] Baylis D., Bartlett D.B., Syddall H.E., Ntani G., Gale C.R., Cooper C., Lord J.M., Sayer A.A. (2013). Immune-endocrine biomarkers as predictors of frailty and mortality: A 10-year longitudinal study in community-dwelling older people. Age.

[9] Yanagita I., Fujihara Y., Eda T., Tajima M., Yonemura K., Kawajiri T., Yamaguchi N., Asakawa H., Nei Y., Kayashima Y. (2018). Low glycated hemoglobin level is associated with severity of frailty in Japanese elderly diabetes patients. J. Diabetes Investig..

[10] Barzilay J.I., Blaum C., Moore T., Xue Q.L., Hirsch C.H., Walston J.D., Fried L.P. (2007). Insulin resistance and inflammation as precursors of frailty: The Cardiovascular Health Study. Arch. Intern. Med..

[11] Soysal P., Stubbs B., Lucato P., Luchini C., Solmi M., Peluso R., Sergi G., Isik A.T., Manzato E., Maggi S. (2016). Inflammation and frailty in the elderly: A systematic review and meta-analysis. Ageing Res. Rev..

[12] Boxer R.S., Dauser D.A., Walsh S.J., Hager W.D., Kenny A.M. (2008). The association between vitamin D and inflammation with the 6-minute walk and frailty in patients with heart failure. J. Am. Geriatr. Soc..

[13] McKechnie D.G.J., Papacosta A.O., Lennon L.T., Ramsay S.E., Whincup P.H., Wannamethee S.G. (2021). Associations between inflammation, cardiovascular biomarkers and incident frailty: The British Regional Heart Study. Age Ageing.

[14] Xu Y., Wang M., Chen D., Jiang X., Xiong Z. (2022). Inflammatory biomarkers in older adults with frailty: A systematic review and meta-analysis of cross-sectional studies. Aging Clin. Exp. Res..

[15] Picca A., Coelho-Junior H.J., Calvani R., Marzetti E., Vetrano D.L. (2022). Biomarkers shared by frailty and sarcopenia in older adults: A systematic review and meta-analysis. Ageing Res. Rev..

[16] Ruangsuriya J., Siviroj P., Ayood P., Ongprasert K., Kaewtunjai N., Srichairatanakool S., Tuntiwechapikul W., Chittrakul J. (2019). Biomarker: Trolox equivalent antioxidant capacity and telomere length of Thai elderly people with frailty. J. Health Sci..

[17] Zaslavsky O., Walker R.L., Crane P.K., Gray S.L., Larson E.B. (2016). Glucose Levels and Risk of Frailty. J. Gerontol. Ser. A.

[18] Collins R. (2012). What makes UK Biobank special?. Lancet.

[19] Fried L.P., Tangen C.M., Walston J., Newman A.B., Hirsch C., Gottdiener J., Seeman T., Tracy R., Kop W.J., Burke G. (2001). Frailty in Older Adults: Evidence for a Phenotype. J. Gerontol. Ser. A.

[20] Petermann-Rocha F., Gray S.R., Pell J.P., Ho F.K., Celis-Morales C. (2021). The joint association of sarcopenia and frailty with incidence and mortality health outcomes: A prospective study. Clin. Nutr..

[21] Elliott P., Peakman T.C., UK Biobank (2008). The UK Biobank sample handling and storage protocol for the collection, processing and archiving of human blood and urine. Int. J. Epidemiol..

[22] Petermann-Rocha F., Gray S.R., Pell J.P., Celis-Morales C., Ho F.K. (2020). Biomarkers Profile of People With Sarcopenia: A Cross-sectional Analysis From UK Biobank. J. Am. Med. Dir. Assoc..

[23] Lees J.S., Welsh C.E., Celis-Morales C.A., Mackay D., Lewsey J., Gray S.R., Lyall D.M., Cleland J.G., Gill J.M.R., Jhund P.S. (2019). Glomerular filtration rate by differing measures, albuminuria and prediction of cardiovascular disease, mortality and end-stage kidney disease. Nat. Med..

[24] Pillatt A.P., Silva B.D., Franz L.B.B., Berlezi E.M., Schneider R.H. (2021). Muscle, endocrine, and immunological markers of frailty in older people. Exp. Gerontol..

[25] Mohr B.A., Bhasin S., Kupelian V., Araujo A.B., O’Donnell A.B., McKinlay J.B. (2007). Testosterone, sex hormone-binding globulin, and frailty in older men. J. Am. Geriatr. Soc..

[26] Dalrymple L.S., Katz R., Rifkin D.E., Siscovick D., Newman A.B., Fried L.F., Sarnak M.J., Odden M.C., Shlipak M.G. (2013). Kidney function and prevalent and incident frailty. Clin. J. Am. Soc. Nephrol..

[27] Toth M.J., Matthews D.E., Tracy R.P., Previs M.J. (2005). Age-related differences in skeletal muscle protein synthesis: Relation to markers of immune activation. Am. J. Physiol. Endocrinol. Metab..

[28] Motta F., Sica A., Selmi C. (2020). Frailty in Rheumatic Diseases. Front. Immunol..

[29] Kang S.H., Do J.Y., Kim J.C. (2021). Association Between Alkaline Phosphatase and Muscle Mass, Strength, or Physical Performance in Patients on Maintenance Hemodialysis. Front. Med..

[30] Le Couteur D.G., Blyth F.M., Creasey H.M., Handelsman D.J., Naganathan V., Sambrook P.N., Seibel M.J., Waite L.M., Cumming R.G. (2010). The association of alanine transaminase with aging, frailty, and mortality. J. Gerontol. Ser. A Biol. Sci. Med. Sci..

[31] Nishikawa H., Enomoto H., Yoh K., Iwata Y., Sakai Y., Kishino K., Ikeda N., Takashima T., Aizawa N., Takata R. (2019). Combined Albumin-Bilirubin Grade and Skeletal Muscle Mass as a Predictor in Liver Cirrhosis. J. Clin. Med..

[32] Yanagita I., Fujihara Y., Iwaya C., Kitajima Y., Tajima M., Honda M., Teruya Y., Asakawa H., Ito T., Eda T. (2020). Low serum albumin, aspartate aminotransferase, and body mass are risk factors for frailty in elderly people with diabetes–A cross-sectional study. BMC Geriatr..

[33] Stewart R. (2019). Cardiovascular Disease and Frailty: What Are the Mechanistic Links?. Clin. Chem..

[34] Chan M.S., Arnold M., Offer A., Hammami I., Mafham M., Armitage J., Perera R., Parish S. (2021). A Biomarker-based Biological Age in UK Biobank: Composition and Prediction of Mortality and Hospital Admissions. J. Gerontol. A Biol. Sci. Med. Sci..

[35] Lv Y.B., Mao C., Gao X., Yin Z.X., Kraus V.B., Yuan J.Q., Zhang J., Luo J.S., Zeng Y., Shi X.M. (2019). Triglycerides Paradox Among the Oldest Old: “The Lower the Better?”. J. Am. Geriatr. Soc..

[36] Ballew S.H., Chen Y., Daya N.R., Godino J.G., Windham B.G., McAdams-DeMarco M., Coresh J., Selvin E., Grams M.E. (2017). Frailty, Kidney Function, and Polypharmacy: The Atherosclerosis Risk in Communities (ARIC) Study. Am. J. Kidney Dis..

[37] Beasley J.M., LaCroix A.Z., Neuhouser M.L., Huang Y., Tinker L., Woods N., Michael Y., Curb J.D., Prentice R.L. (2010). Protein intake and incident frailty in the Women’s Health Initiative observational study. J. Am. Geriatr. Soc..

[38] Morris R., Carstairs V. (1991). Which deprivation? A comparison of selected deprivation indexes. J. Public Health Med..

[39] Guo W., Key T.J., Reeves G.K. (2019). Accelerometer compared with questionnaire measures of physical activity in relation to body size and composition: A large cross-sectional analysis of UK Biobank. BMJ Open.

[40] Kyle S.D., Sexton C.E., Feige B., Luik A.I., Lane J., Saxena R., Anderson S.G., Bechtold D.A., Dixon W., Little M.A. (2017). Sleep and cognitive performance: Cross-sectional associations in the UK Biobank. Sleep Med..

[41] Bradbury K.E., Young H.J., Guo W., Key T.J. (2018). Dietary assessment in UK Biobank: An evaluation of the performance of the touchscreen dietary questionnaire. J. Nutr. Sci..

[42] Barnett K., Mercer S.W., Norbury M., Watt G., Wyke S., Guthrie B. (2012). Epidemiology of multimorbidity and implications for health care, research, and medical education: A cross-sectional study. Lancet.

[43] Jani B.D., McQueenie R., Nicholl B.I., Field R., Hanlon P., Gallacher K.I., Mair F.S., Lewsey J. (2021). Association between patterns of alcohol consumption (beverage type, frequency and consumption with food) and risk of adverse health outcomes: A prospective cohort study. BMC Med..

[44] Carey E.J., Steidley D.E., Aqel B.A., Byrne T.J., Mekeel K.L., Rakela J., Vargas H.E., Douglas D.D. (2010). Six-minute walk distance predicts mortality in liver transplant candidates. Liver Transplant..

[45] Kameda M., Teruya T., Yanagida M., Kondoh H. (2021). Reduced uremic metabolites are prominent feature of sarcopenia, distinct from antioxidative markers for frailty. Aging.

[46] Liu X., Chen X., Hu F., Xia X., Hou L., Zhang G., Peng X., Sun X., Luo S., Yue J. (2022). Higher uric acid serum levels are associated with sarcopenia in west China: A cross-sectional study. BMC Geriatr..

[47] Hyde Z., Flicker L., Almeida O.P., Hankey G.J., McCaul K.A., Chubb S.A., Yeap B.B. (2010). Low free testosterone predicts frailty in older men: The health in men study. J. Clin. Endocrinol. Metab..

[48] Bruyère O., Cavalier E., Buckinx F., Reginster J.Y. (2017). Relevance of vitamin D in the pathogenesis and therapy of frailty. Curr. Opin. Clin. Nutr. Metab. Care.

[49] Landi F., Russo A., Pahor M., Capoluongo E., Liperoti R., Cesari M., Bernabei R., Onder G. (2008). Serum high-density lipoprotein cholesterol levels and mortality in frail, community-living elderly. Gerontology.

[50] Bonnefoy M., Abidi H., Jauffret M., Garcia I., Surrace J.P., Drai J. (2002). Hypocholesterolemia in hospitalized elderly: Relations with inflammatory and nutritional status. Rev. Med. Interne.

[51] Nordestgaard B.G., Chapman M.J., Ray K., Borén J., Andreotti F., Watts G.F., Ginsberg H., Amarenco P., Catapano A., Descamps O.S. (2010). Lipoprotein(a) as a cardiovascular risk factor: Current status. Eur. Heart J..

[52] Taskinen M.R., Barter P.J., Ehnholm C., Sullivan D.R., Mann K., Simes J., Best J.D., Hamwood S., Keech A.C. (2010). Ability of traditional lipid ratios and apolipoprotein ratios to predict cardiovascular risk in people with type 2 diabetes. Diabetologia.

[53] Cicek H., Bayil S., Zer Y., Celik A., Geyikli I. (2007). Comparison of Lipoprotein(a) levels between elderly and middle-aged men with coronary artery disease. Ann. N. Y. Acad. Sci..

[54] Cohen A.A., Legault V., Fuellen G., Fülöp T., Fried L.P., Ferrucci L. (2018). The risks of biomarker-based epidemiology: Associations of circulating calcium levels with age, mortality, and frailty vary substantially across populations. Exp. Gerontol..

