# Supplementary material for: Identifying the Biomarker Profile of Pre-Frail and Frail People: A Cross-Sectional Analysis from UK Biobank
Source: Int J Environ Res Public Health. 2023 Jan 29;20(3):2421. doi: 10.3390/ijerph20032421 (PMC9915970; doi:10.3390/ijerph20032421)
Supplement: Supplementary file 1 [file ijerph-20-02421-s001.zip › ijerph-2147202-supplementary.pdf]

Supplementary Material

Identifying the biomarker profile of pre-frail and frail people: a cross-sectional analysis from UK Biobank

Table S1. Modified frailty criteria. 2
Table S2. Cut-off points for grip strength criterion for frailty. 3
Table S3. Biomarkers by frailty status (Women). 4
Table S4. Biomarkers by frailty status (Men). 5
Table S5. Associations between biomarkers and frailty categories in women. 6
Table S6. Associations between biomarkers and frailty categories in men 7

Table S1. Modified frailty criteria.

Indicators	Frailty (touch-screen questionnaire)
Weight loss	Self-reported: ''Compared with one year ago, has your weight changed?'' (response: yes=lost weight; no=another option)
Exhaustion	Self-reported: "Over the past two weeks, how often have you felt tired or had little energy?'' (response: yes=more than half time or nearly every day; no=another option)
Physical activity	IPAQ short form was used to collect the total physical activity (MET-hours/week). The lowest quintile (20%) was defined as fulfilling the low physical activity level for frailty.
Gait speed	Self-reported: "How would you describe your usual walking pace?" (response: yes=slow pace, no=average or brisk pace)
Grip strength	Hand-grip strength measurements were performed using a Jamar J00105 hydraulic hand dynamometer. Cut-off points for grip strength (Kg) were adjusted by sex and BMI, and were derived from Fried and colleagues.

Criteria were adapted from Fried and colleagues and these adaptations were based on previous paper.[14, 15]

Table S2. Cut-off points for grip strength criterion for frailty.

BMI (Kg/m²)	Grip strength (Kg)
Men	
≤24	≤29
24.1-26	≤30
26.1-28	≤30
>28	≤32
Women	
≤23	≤17
23.1-26	≤17.3
26.1-29	≤18
>29	≤21

Cut-off points were adjusted by sex and BMI.[10]

Table S3. Biomarkers by frailty status (Women).

	No Frail	Pre-frail	Frail
Albumin (g/l), mean (95%CI)	45.0 (45.0; 45.1)	44.9 (44.8; 44.9)	44.6 (44.6; 44.7)
Glucose (mmol/l), mean (95%CI)	5.06 (5.05; 5.07)	5.05 (5.05; 5.06)	5.15 (5.13; 5.18)
C-reactive protein (mg/l), mean (95%CI)	2.4 (2.4; 2.5)	2.6 (2.6; 2.7)	3.6 (3.5; 3.7)
HDL cholesterol (mmol/l), mean (95%CI)	1.59 (1.58; 1.59)	1.56 (1.56; 1.56)	1.55 (1.54; 1.56)
LDL cholesterol (mmol/l), mean (95%CI)	3.65 (3.64; 3.65)	3.62 (3.62; 3.63)	3.55 (3.53; 3.57)
ALP (U/L), mean (95%CI)	84.4 (84.2; 84.6)	85.6 (85.4; 85.7)	87.2 (86.5; 87.8)
ALT (U/L), mean (95%CI)	19.9 (19.8; 20.0)	20.0 (19.9; 20.1)	19.7 (19.4; 20.0)
Apolipoprotein A1 (g/L), mean (95%CI)	1.63 (1.63; 1.63)	1.61 (1.61; 1.61)	1.60 (1.59; 1.61)
Apolipoprotein B (g/L), mean (95%CI)	1.04 (1.04; 1.04)	1.04 (1.04; 1.04)	1.02 (1.02; 1.03)
AST (U/L), mean (95%CI)	24.6 (24.5; 24.6)	24.3 (24.3; 24.4)	24.4 (24.1; 24.6)
GGT (U/L), mean (95%CI)	27.6 (27.3; 27.8)	28.7 (28.5; 28.9)	32.4 (31.7; 33.1)
Direct bilirubin (µmol/L), mean (95%CI)	1.51 (1.51; -1.52)	1.49 (1.49; 1.50)	1.51 (1.49; 1.53)
Urea (mmol/L), mean (95%CI)	5.23 (5.22; 5.24)	5.23 (5.22; 5.24)	5.27 (5.24; 5.30)
Lipoprotein A (nmol/L), mean (95%CI)	53.1 (52.6; 53.7)	53.2 (52.8; 53.7)	50.7 (49.2; 52.3)
Oestradiol (pmol/L), mean (95%CI)	141.7 (139.0; 144.4)	137.0 (134.5; 139.5)	135.7 (127.6; 143.8)
Phosphate (mmol/L), mean (95%CI)	1.19 (1.19; 1.20)	1.20 (1.20; 1.20)	1.21 (1.21; 1.22)
Rheumatoid factor (IU/ml), mean (95%CI)	5.4 (5.3; 5.5)	5.8 (5.7; 5.9)	6.5 (6.2; 6.8)
SHBG (nmol/L), mean (95%CI)	63.8 (63.5; 64.1)	63.6 (63.4; 63.9)	66.0 (65.2; 66.8)
Total bilirubin (µmol/L), mean (95%CI)	8.2 (8.2; 8.3)	8.1 (8.0; 8.1)	8.0 (7.9; 8.1)
Testosterone (nmol/L), mean (95%CI)	1.03 (1.02; 1.03)	1.02 (1.01; 1.02)	0.98 (0.97; 1.00)
Total protein (g/L), mean (95%CI)	72.5 (72.5; 72.5)	72.4 (72.4; 72.4)	72.2 (72.1; 72.3)
Triglycerides (mmol/L), mean (95%CI)	1.53 (1.52; 1.54)	1.55 (1.54; 1.55)	1.58 (1.56; 1.60)
Urate (µmol/L), mean (95%CI)	266.3 (265.9; 266.8)	267.1 (266.7; 267.6)	267.9 (266.5; 269.3)
Vitamin D (nmol/L), mean (95%CI)	49.5 (49.4; 49.7)	48.4 (48.2; 48.5)	47.0 (46.5; 47.5)
Calcium (mmol/L), mean (95%CI)	2.389 (2.388; 2.390)	2.385 (2.385; 2.386)	2.381 (2.378; 2.384)
Total cholesterol (mmol/l), mean (95%CI)	5.89 (5.88; 5.89)	5.84 (5.83; 5.85)	5.75 (5.73; 5.78)
Creatinine (µmol/L), mean (95%CI)	64.9 (64.8; 65.1)	64.1 (64.0; 64.2)	64.2 (63.9; 64.6)
Cystatin C (mg/L), mean (95%95%CI)	0.87 (0.87; 0.87)	0.88 (0.88; 0.88)	0.91 (0.91; 0.92)
IGF-1 (nmol/L), mean (95%CI)	21.2 (21.2; 21.3)	21.1 (21.1; 21.1)	20.5 (20.4; 20.7)
HbA1c (mmol/l), mean (95%CI)	35.90 (35.85; 35.94)	35.86 (35.82; 35.90)	36.55 (36.42; 36.68)
eGFR, mean (95%CI)	95.5 (95.4; 95.6)	94.6 (94.5; 94.7)	92.3 (92.0; 92.7)

Difference between groups was estimated using linear regression analyses. All analyses were adjusted for age, deprivation, BMI, smoking status, sleeping time, total sedentary time, morbidity count, medication, and dietary intake (alcohol, red meat, processed meat, fruit and vegetable intake).

Table S4. Biomarkers by frailty status (Men).

	No-frail (95% CI)	Pre-frail (95% CI)	Frail (95% CI)
Albumin (g/l), mean (95%CI)	45.5 (45.5; 45.5)	45.4 (45.4; 45.5)	44.9 (44.8; 45.0)
Glucose (mmol/l), mean (95%CI)	5.12 (5.11; 5.14)	5.19 (5.18; 5.21)	5.51 (5.46; 5.57)
C-reactive protein (mg/l), mean (95%CI)	2.2 (2.1; 2.3)	2.4 (2.4; 2.5)	3.7 (3.5; 3.9)
HDL cholesterol (mmol/l), mean (95%CI)	1.23 (1.22; 1.23)	1.20 (1.20; 1.21)	1.18 (1.17; 1.19)
LDL cholesterol (mmol/l), mean (95%CI)	3.44 (3.44; 3.45)	3.40 (3.39; 3.41)	3.30 (3.27; 3.33)
ALP (U/L), mean (95%CI)	82.4 (82.1; 82.7)	83.2 (82.9; 83.5)	87.5 (86.5; 88.5)
ALT (U/L), mean (95%CI)	26.25 (26.09; 26.41)	26.18 (26.02; 26.34)	24.54 (24.0; 25.09)
Apolipoprotein A1 (g/L), mean (95%CI)	1.38 (1.38; 1.39)	1.36 (1.36; 1.37)	1.34 (1.33; 1.35)
Apolipoprotein B (g/L), mean (95%CI)	1.01 (1.01; 1.02)	1.00 (1.00; 1.01)	0.98 (0.98; 0.99)
AST (U/L), mean (95%CI)	27.9 (27.8; 28.0)	27.3 (27.2; 27.4)	26.3 (25.9; 26.7)
GGT (U/L), mean (95%CI)	37.1 (36.6; 37.5)	38.1 (37.7; 38.5)	43.2 (41.7; 44.7)
Direct bilirubin (μmol/L), mean (95%CI)	1.95 (1.94; 1.97)	1.95 (1.94; 1.97)	1.99 (1.95; 2.03)
Urea (mmol/L), mean (95%CI)	5.7 (5.7; 5.7)	5.7 (5.7; 5.7)	5.9 (5.9; 6.0)
Lipoprotein A (nmol/L), mean (95%CI)	47.3 (46.6; 48.0)	46.9 (46.3; 47.6)	48.4 (46.1; 50.7)
Oestradiol (pmol/L), mean (95%CI)	31.9 (31.2; 32.7)	32.8 (32.0; 33.5)	32.4 (29.8; 35.1)
Phosphate (mmol/L), mean (95%CI)	1.11 (1.11; 1.12)	1.12 (1.12; 1.13)	1.14 (1.13; 1.14)
Rheumatoid factor (IU/ml), mean (95%CI)	5.3 (5.1; 5.4)	5.3 (5.2; 5.5)	6.0 (5.6; 6.5)
SHBG (nmol/L), mean (95%CI)	41.3 (41.1; 41.5)	40.9 (40.7; 41.1)	41.8 (41.1; 42.4)
Total bilirubin (μmol/L), mean (95%CI)	10.5 (10.4; 10.5)	10.4 (10.3; 10.4)	10.1 (9.9; 10.3)
Testosterone (nmol/L), mean (95%CI)	12.2 (12.2; 12.3)	12.0 (12.0; 12.0)	11.5 (11.3; 11.6)
Total protein (g/L), mean (95%CI)	72.5 (72.5; 72.6)	72.5 (72.4; 72.5)	72.3 (72.1; 72.5)
Triglycerides (mmol/L), mean (95%CI)	1.90 (1.89; 1.91)	1.93 (1.92; 1.95)	1.96 (1.92; 2.00)
Urate (μmol/L), mean (95%CI)	341.7 (340.9; 342.5)	341.4 (340.6; 342.1)	340.0 (337.3; 342.5)
Vitamin D (nmol/L), mean (95%CI)	49.3 (49.1; 49.6)	47.3 (47.1; 47.5)	43.9 (43.1; 44.7)
Calcium (mmol/L), mean (95%CI)	2.370 (2.369; 2.370)	2.369 (2.368; 2.370)	2.359 (2.356; 2.363)
Total cholesterol (mmol/l), mean (95%CI)	5.38 (5.36; 5.39)	5.30 (5.29; 5.31)	5.18 (5.14; 5.22)
Creatinine (μmol/L), mean (95%CI)	82.8 (82.6; 83.1)	82.2 (82.0; 82.5)	85.5 (84.7; 86.4)
Cystatin C (mg/L), mean (95%CI)	0.95 (0.94; 0.95)	0.96 (0.96; 0.96)	1.04 (1.03; 1.05)
IGF-1 (nmol/L), mean (95%CI)	22.4 (22.3; 22.4)	22.4 (22.4; 22.5)	22.1 (21.9; 22.3)
HbA1c (mmol/l), mean (95%CI)	36.5 (36.4; 36.6)	36.9 (36.8; 37.0)	38.8 (38.5; 39.1)
eGFR, mean (95%CI)	87.2 (87.0; 87.4)	86.2 (86.0; 86.3)	82.0 (81.4; 82.5)

Difference between groups was estimated using linear regression analyses. All analyses were adjusted for age, deprivation, BMI, smoking status, sleeping time, total sedentary time, morbidity count, medication, and dietary intake (alcohol, red meat, processed meat, fruit and vegetable intake).

Table S5. Associations between biomarkers and frailty categories in women.

	No Frail<br>β (95% CI)	Pre-frail<br>β (95% CI)	Frail<br>β (95% CI)
Albumin	1.00 (Ref.)	-0.06 (-0.07; -0.05)	-0.11 (-0.14; -0.09)
ALP	1.00 (Ref.)	0.04 (0.02; 0.05)	0.07 (0.04; 0.09)
ALT	1.00 (Ref.)	0.01 (-0.004; 0.01)	-0.02 (-0.05; -0.002)
Apolipoprotein A1	1.00 (Ref.)	-0.08 (-0.09; -0.07)	-0.10 (-0.12; -0.07)
Apolipoprotein B	1.00 (Ref.)	-0.01 (-0.02; -0.0008)	-0.08 (-0.10; -0.05)
AST	1.00 (Ref.)	-0.02 (-0.03; -0.01)	-0.03 (-0.05; -0.003)
Direct bilirubin	1.00 (Ref.)	-0.02 (-0.03; -0.01)	0.004 (-0.02; 0.02)
Urea	1.00 (Ref.)	0.005 (-0.006; 0.01)	0.04 (0.01; 0.06)
Calcium	1.00 (Ref.)	-0.03 (-0.05; -0.02)	-0.07 (-0.10; -0.04)
Cholesterol	1.00 (Ref.)	-0.04 (-0.05; -0.03)	-0.11 (-0.13; -0.08)
Creatinine	1.00 (Ref.)	-0.04 (-0.05; -0.04)	-0.04 (-0.06; -0.02)
Cystatin C	1.00 (Ref.)	0.04 (0.03; 0.05)	0.22 (0.20; 0.24)
GGT	1.00 (Ref.)	0.02 (0.01; 0.03)	0.09 (0.07; 0.11)
Glucose	1.00 (Ref.)	-0.006 (-0.02; 0.004)	0.07 (0.04; 0.09)
HbA1c	1.00 (Ref.)	-0.008 (-0.02; 0.001)	0.08 (0.06; 0.10)
HDL cholesterol	1.00 (Ref.)	-0.07 (-0.08; -0.06)	-0.08 (-0.10; -0.05)
IGF-1	1.00 (Ref.)	-0.02 (-0.03; -0.006)	-0.09 (-0.12; -0.07)
LDL cholesterol	1.00 (Ref.)	-0.02 (-0.03; -0.01)	-0.10 (-0.12; -0.08)
Lipoprotein A	1.00 (Ref.)	0.002 (-0.01; 0.01)	-0.04 (-0.07; -0.02)
Oestradiol	1.00 (Ref.)	-0.02 (-0.03; -0.004)	-0.03 (-0.06; 0.006)
Phosphate	1.00 (Ref.)	0.04 (0.03; 0.06)	0.12 (0.09; 0.14)
Rheumatoid factor	1.00 (Ref.)	0.03 (0.02; 0.04)	0.08 (0.05; 0.11)
SHBG	1.00 (Ref.)	-0.006 (-0.02; 0.007)	0.08 (0.05; 0.11)
Total bilirubin	1.00 (Ref.)	-0.03 (-0.04; -0.02)	-0.04 (-0.06; -0.01)
Testosterone	1.00 (Ref.)	-0.002 (-0.003; -0.0005)	-0.007 (-0.009; -0.004)
Total protein	1.00 (Ref.)	-0.03 (-0.04; -0.02)	-0.09 (-0.12; -0.06)
Triglycerides	1.00 (Ref.)	0.01 (0.004; 0.02)	0.04 (0.02; 0.06)
Urate	1.00 (Ref.)	0.008 (-0.0003; 0.02)	0.008 (-0.01; 0.03)
Vitamin D	1.00 (Ref.)	-0.06 (-0.07; -0.05)	-0.12 (-0.15; -0.10)
eGFR	1.00 (Ref.)	-0.05 (-0.06; -0.04)	-0.17 (-0.19; -0.15)

Data presented as β-coefficient and its 95% CI. Non-frail individuals were considered as the reference group in each case. All analyses were adjusted for age, deprivation, BMI, smoking status, sleeping time, total sedentary time, morbidity count, medication, dietary intake (alcohol, red meat, processed meat, fruit and vegetable intake) and CRP.

Table S6. Associations between biomarkers and frailty categories in men.

	No Frail<br>β (95% CI)	Pre-frail<br>β (95% CI)	Frail<br>β (95% CI)
Albumin	1.00 (Ref.)	-0.02 (-0.03; -0.001)	-0.18 (-0.23; -0.14)
ALP	1.00 (Ref.)	0.02 (0.008; 0.04)	0.14 (0.10; 0.18)
ALT	1.00 (Ref.)	-0.004 (-0.02; 0.01)	-0.11 (-0.15; -0.08)
Apolipoprotein A1	1.00 (Ref.)	-0.07 (-0.08; -0.06)	-0.14 (-0.17; -0.11)
Apolipoprotein B	1.00 (Ref.)	-0.04 (-0.05; -0.02)	-0.12 (-0.16; -0.09)
AST	1.00 (Ref.)	-0.05 (-0.07; -0.04)	-0.14 (-0.18; -0.11)
Direct bilirubin	1.00 (Ref.)	0.001 (-0.02; 0.02)	0.04 (-0.01; 0.09)
Urea	1.00 (Ref.)	-0.02 (-0.04; -0.003)	0.17 (0.12; 0.21)
Calcium	1.00 (Ref.)	-0.01 (-0.03; 0.01)	-0.10 (-0.14; -0.06)
Cholesterol	1.00 (Ref.)	-0.06 (-0.08; -0.05)	-0.16 (-0.19; -0.12)
Creatinine	1.00 (Ref.)	-0.04 (-0.05; -0.02)	0.13 (0.08; 0.18)
Cystatin C	1.00 (Ref.)	0.07 (0.05; 0.09)	0.50 (0.45; 0.54)
GGT	1.00 (Ref.)	0.02 (0.01; 0.03)	0.11 (0.07; 0.15)
Glucose	1.00 (Ref.)	0.06 (0.04; 0.07)	0.30 (0.25; 0.35)
HbA1c	1.00 (Ref.)	0.05 (0.04; 0.07)	0.33 (0.28; 0.37)
HDL cholesterol	1.00 (Ref.)	-0.06 (-0.07; -0.05)	-0.10 (-0.13; -0.07)
IGF-1	1.00 (Ref.)	0.01 (-0.003; 0.03)	-0.04 (-0.08; 0.01)
LDL cholesterol	1.00 (Ref.)	-0.05 (-0.07; -0.04)	-0.15 (-0.19; -0.12)
Lipoprotein A	1.00 (Ref.)	-0.01 (-0.02; 0.01)	0.01 (-0.03; 0.05)
Oestradiol	1.00 (Ref.)	0.003 (-0.001; 0.01)	0.0002 (-0.01; 0.01)
Phosphate	1.00 (Ref.)	0.06 (0.04; 0.07)	0.14 (0.09; 0.18)
Rheumatoid factor	1.00 (Ref.)	0.004 (-0.01; 0.02)	0.06 (0.02; 0.09)
SHBG	1.00 (Ref.)	-0.01 (-0.02; -0.004)	0.03 (0.002; 0.05)
Total bilirubin	1.00 (Ref.)	-0.02 (-0.04; -0.003)	-0.07 (-0.11; -0.02)
Testosterone	1.00 (Ref.)	-0.04 (-0.05; -0.03)	-0.11 (-0.14; -0.09)
Total protein	1.00 (Ref.)	-0.01 (-0.03; 0.01)	-0.08 (-0.12; -0.03)
Triglycerides	1.00 (Ref.)	0.03 (0.02; 0.05)	0.07 (0.03; 0.11)
Urate	1.00 (Ref.)	-0.01 (-0.02; 0.01)	-0.03 (-0.06; 0.003)
Vitamin D	1.00 (Ref.)	-0.09 (-0.11; -0.08)	-0.26 (-0.30; -0.22)
eGFR	1.00 (Ref.)	-0.06 (-0.07; -0.04)	-0.28 (-0.31; -0.24)

Data presented as β-coefficient and its 95% CI. Non-frail individuals were considered as the reference group in each case. All analyses were adjusted for age, deprivation, BMI, smoking status, sleeping time, total sedentary time, morbidity count, medication, dietary intake (alcohol, red meat, processed meat, fruit and vegetable intake) and CRP.
